# Genetics, epigenetics and transgenerational transmission of obesity in children

**DOI:** 10.3389/fendo.2022.1006008

**Published:** 2022-11-14

**Authors:** Nadia Panera, Claudia Mandato, Annalisa Crudele, Sara Bertrando, Pietro Vajro, Anna Alisi

**Affiliations:** ^1^ Unit of Molecular Genetics of Complex Phenotypes, Bambino Gesù Children’s Hospital, IRCCS, Rome, Italy; ^2^ Pediatrics Section, Department of Medicine, Surgery and Dentistry “Scuola Medica Salernitana”, University of Salerno, Baronissi, Salermo, Italy; ^3^ Pediatrics Clinic, San Giovanni di Dio e Ruggi d’Aragona University Hospital, Salerno, Italy

**Keywords:** obesity, pregnancy, gestation, transgenerational transmission, genetics, epigenetics, polymorphisms, methylation

## Abstract

Sedentary lifestyle and consumption of high-calorie foods have caused a relentless increase of overweight and obesity prevalence at all ages. Its presently epidemic proportion is disquieting due to the tight relationship of obesity with metabolic syndrome and several other comorbidities which do call for urgent workarounds. The usual ineffectiveness of present therapies and failure of prevention campaigns triggered overtime a number of research studies which have unveiled some relevant aspects of obesity genetic and epigenetic inheritable profiles. These findings are revealing extremely precious mainly to serve as a likely extra arrow to allow the clinician’s bow to achieve still hitherto unmet preventive goals. Evidence now exists that maternal obesity/overnutrition during pregnancy and lactation convincingly appears associated with several disorders in the offspring independently of the transmission of a purely genetic predisposition. Even the pre-conception direct exposure of either father or mother gametes to environmental factors can reprogram the epigenetic architecture of cells. Such phenomena lie behind the transfer of the obesity susceptibility to future generations through a mechanism of epigenetic inheritance. Moreover, a growing number of studies suggests that several environmental factors such as maternal malnutrition, hypoxia, and exposure to excess hormones and endocrine disruptors during pregnancy and the early postnatal period may play critical roles in programming childhood adipose tissue and obesity. A deeper understanding of how inherited genetics and epigenetics may generate an obesogenic environment at pediatric age might strengthen our knowledge about pathogenetic mechanisms and improve the clinical management of patients. Therefore, in this narrative review, we attempt to provide a general overview of the contribution of heritable genetic and epigenetic patterns to the obesity susceptibility in children, placing a particular emphasis on the mother-child dyad.

## Introduction

The steadily increasing prevalence of obesity in the general population it has now become a significant burden for human health (1). Worldwide estimates revealed more than 7% of children and adolescents had obesity in 2016 compared with less than 1% in 1975 (2). According to global health estimates provided by World Health Organization (WHO) 38.2 million of children under the age of 5 years were overweight or obese in 2019. Data also indicates that the prevalence of overweight and obesity in children and adolescents (from 5 to 19 years) has increased from approximately 4% in 1975 to more than 18% in 2016 noting that no significant differences emerge between girls and boys (3).

Obesity is a multifaceted disease widely regarded by now as the main risk condition for developing the metabolic syndrome in both children and adults (4, 5). It is precisely for this aspect that obesity is closely associated with a series of pathological manifestations such as insulin resistance, Type 2 diabetes (T2D) and non-alcoholic fatty liver disease (NAFLD) and sleep-related breathing disorder such as obstructive sleep apnea (OSA) (6, 7). In particular, over the past two decades, the mechanistic exploration of the nexus between obesity levels and metabolic diseases has highlighted a close relationship between nutrient excess, genetics, epigenetics, oxidative stress, and inflammatory signals (8, 9). It is widely accepted as a fact that the epidemic proportion reached by the obesity in pediatric population is mostly a consequence of the sedentary lifestyle and consumption of high-calorie junk foods, clearly indicating the crucial role of the current obesogenic environment in favoring obesity’s and comorbidities’ epidemics (10). However, the well-documented individual variation in body weight, central adiposity, and particularly the differences in the onset and severity of the obesity-associated metabolic diseases, has forced the researchers to search other driving causes, such as the genetic and epigenetic inheritable profiles, even if these mechanisms have not yet been fully explored (11). It is known that direct exposures of ancestral germline (sperm and eggs) to environmental factors and altered nutrition can reprogram the epigenome of the cells, thus transmitting the susceptibility for the obesity to future generations through a mechanism of epigenetic transgenerational inheritance (12). Moreover, maternal obesity is currently being studied to clarify the links between maternal overnutrition during gestation and breastfeeding period, and the occurrence of hepatic/metabolic/cardiovascular disorders in the resultant offspring independently of genetic predisposition. According to an increasing number of studies, environmental factors such as maternal malnutrition, hypoxia and exposure to excess hormones and endocrine disruptors, during gestation and early postnatal period, play a decisive role in the programming of adipose tissue and obesity in the childhood (13).

In this review, we provide an overview of contribution of heritable genetic and epigenetic causes in development of obesity in childhood and focus on the role of epigenetic transgenerational inheritance on the obesity susceptibility.

## Methods

Here, we have summarized the existing literature that could be relevant to the purpose of our narrative review. Since we focused on the impact of heritable genetics and epigenetics factors on pediatric obesity we structured the article by two main subjects, including the role of gene mutations and epigenetic modifications. For these topics, literature search was performed in the PubMed and Scopus Databases with the following search terms: “polygenic obesity” OR “monogenic obesity”, AND “children”; “epigenetic”, AND “obesity”, AND “children”. We also reported the effects of parental and *in utero* epigenetic mechanisms on the obese phenotype and its comorbidities by extrapolating from the main search on transgenerational transmission of obesity. We searched for clinical studies, as well as reviews, published in the English language, until June of 2022. Eligible papers for our narrative review included studies that reported convincing data and theories supporting the hypothesis of genetics and epigenetics inheritance of obesity.

## Genetics of pediatric obesity

Obesity is a multifactorial disease where environmental factors and several genes play important pathogenetic roles (14). Indeed, excessive fat accumulation due to an excess intake of sugar-sweetened beverages, high-calorie foods, sedentary lifestyle and poor sleeping is a well-known leading cause to obesity (15). In addition, genetics can predispose people to obesity by affecting appetite regulation, body mass index (BMI), metabolism, body-fat distribution and by influencing food preferences and response to exercise (16). Regarding the diagnostic accuracy of BMI to diagnose obesity in children, it has been questioned as it may underestimate the prevalence of adolescents with overweight and obesity (17). Indeed, although obese children and adolescents are around five times more likely to be obese in adulthood than those who were not obese, a large proportion of obese adults (about 70%) were not classed as being obese in childhood or adolescence (17). This effect may be interpreted as due to the effects related to environmental factors linked to developmental origins of health and disease (DOHaD) (18).

Several studies were focused on the identification of genes associated with obesity (19). Indeed, genetic causes of obesity can be classified in different ways including monogenetic, polygenetic, or syndromic obesity (11).

### Monogenic obesity

Monogenetic obesity is rare and involves single-gene mutations or chromosomal deletions and the subjects usually exhibited a severe early-onset obesity associated with hyperphagia and endocrine disorders (20). Most of the gene mutations linked to monogenic obesity have been discovered by experimental studies on transgenic mice (21). Gene mutation are principally located in regions encoding for proteins belonging to the leptin-melanocortin pathway, which is responsible for maintaining energy balance through food consumption and energy expenditure (22) and follows a Mendelian pattern (23). The genetic determinants of monogenic obesity in children have been reported in **Table 1**. In particular, leptin was identified as the product of the obese (ob) gene following the characterization of monogenic obesity (ob/ob) mice model (24). Leptin is an anorexigenic hormone synthesized and secreted by white adipocytes that regulates the food intake and energy balance (25). Concentration of circulating leptin decreases during fasting (26), but increases during refeeding or overfeeding (27). Human congenital leptin deficiency was identified, for the first time, in two obese first-degree cousins from a Pakistani family, where a homozygous frame-shift mutation (c.398delG), leading to a non-secreted truncated leptin protein was genetically characterized (28). However, studies demonstrated that severe obesity develops from rare genetic mutations that affect genes of both leptin and its receptor (29), and lead to congenital leptin deficiency or leptin resistance (30). Other loci which have been implicated in severe childhood monogenic obesity include genes of the proopiomelanocortin (POMC), the melanocortin receptor 4 (MC4R), the protein convertase 1/3 (PC 1/3), the single-minded homolog 1 (SIM1), and the brain-derived neurotrophic factor (BDNF) (31). Obesity caused by mutations in POMC gene shows autosomal recessive inheritance, whereas those in MC4R are autosomal dominant (32). POMC deficiency results in the absence of its cleavage products, and inactivation of MC4R, thus causing hyperphagia, severe obesity, and red hair (33). Mutations in MC4R represent the most common cause of monogenic childhood obesity (34), and more than 150 different nonsynonymous variants of this gene have been described (35). Indeed, rs17782313 and rs12970134 in MC4R gene polymorphisms were associated with obesity in both European and Asian adults and children (36, 37). Also, the mutation in PC 1/3 gene has been correlated with severe early-onset obesity, endocrine disorders, intestinal dysfunctions, and impairment of glucose homeostasis (38, 39). SIM1 gene encode for proteins that play a key role in neuronal development and function of the paraventricular nucleus of hypothalamus which is a crucial region for food intake and control of energy homeostasis (40). Inactivating mutations in this gene have been associated with monogenic obesity in humans and early-onset obesity, although its contribution has only been investigated in a few populations (41, 42). Moreover, few point mutations in SIM1 have been recognized as a cause of monogenic obesity (43, 44). A novel SIM1 variant, p.D134N, potentially linked to monogenic pediatric obesity has been recently identified by Stanikova et al, in a cohort of children and adolescents (45). In addition, dominant forms of non-syndromic monogenic obesity in humans have been associated with mutations in BDNF gene, encoding for the protein involved in proliferation and survival of hypothalamic neurons (46). BDNF is essential for regulating energy balance and food intake and it has been found crucial in leptin down-stream signaling (47). The disruption of one copy of the BDNF gene as consequence of chromosomal rearrangement was described in a child affected by severe obesity and several cognitive alterations (48). While a full functional loss of one BDNF allele seems to cause severe obesity in humans, there is no consistent evidence about the pathogenic variants of this gene. Recent data showed the link of two rare single-nucleotide genetic variants (p.Ile231Val and p.Cys141Gly), to non-syndromic severe early-onset obesity (49). Very recently, BDNF p.Thr2Ile and p.Arg209Gln missense variants were found associated with severe obesity developed during childhood in a 35-year-old and a 46-year-old female patient respectively, thus suggesting a potential obesogenic role of this gene variant (50).

**Table 1:** Genetic determinants of monogenic obesity in children.

GENE NAME	GENE’S PRODUCT FUNCTION	MUTATION	PHENOTYPE	REFERENCE
**Leptin (LEP)**	Food intake and metabolic homeostasis	Homozygous frame-shift mutation; non-secreted truncated leptin protein	Obesity in two Pakistani origin children	[Montague CT, 1997]
**Melanocortin receptor 4 (MC4R)**	Energy homeostasis, food intake and body weight regulator	Heterozygous mutations; Variants: rs17782313, rs12970134	Severe obesity on Dutch children Overweight and obesity in Indian children	[van den Berg L, 2011] [Dwivedi OP, 2013]
**Protein convertase 1/3 (PC 1/3)**	Proteolytic activation of polypeptide hormones and neuropeptides precursors	Missense mutation	Early-onset of severe obesity in an African 6-yr-old boy	[Ranadive SA 2008]
**Single-minded homolog 1 (SIM1)**	Neuronal development and function, food intake and energy homeostasis control	Deletion/inactivating mutation	Early-onset obesity in an American 21-month-old boy	[Gonsalves R, 2020]
**Brain-derived neurotrophic factor (BDNF)**	Proliferation and survival of hypothalamic neurons; energy balance and food intake control	Chromosomal inversion loss-of-function Single-nucleotide *de novo* genetic variants (p.Ile231Val and p.Cys141Gly) p.Thr2Ile and p.Arg209Gln and missense variant	Severe obesity, impaired cognitive function in a 8-yr-old girl. Early-onset of severe obesity in two cases from a Spanish population Severe early-onset obesity in 35-year-old and 46-year-old female Brazilian patient	[Gray J, 2006] [Serra-Juhé C, 2020] [da Fonseca ACP, 2021]

### Polygenic obesity

It is widely accepted that obesity epidemic cannot be traced back to a single gene disorder, but rather to the interaction of a complex genetic background in combination with environment, where single nucleotide polymorphisms (SNPs) may contribute to development of the obese phenotype if they interact with epigenetic inputs. Indeed, polygenic obesity is the most common form of the childhood disease, which results from hundreds of SNPs and observes a pattern of heritability similar to that of other complex traits and diseases (51). Evidence shows that the expression of mutations causing monogenic obesity may, at least in part, be influenced by the individual’s polygenic susceptibility to obesity (52). A genetic study from 573 individuals with severe early onset obesity found 40 very rare predicted functional variants in 13 genes encoding Sema3s (Semaphorin 3 Ligands) and their receptors in severely obese cases compared to controls. Since Sema3 signaling is crucial for gonadotropin-releasing hormone development and function, the study highlighted that the critical perturbations in the development of hypothalamic melanocortin neurons are also crucial for body weight and/or fat mass control (53).

Genome-wide association studies (GWASs) are particularly effective for the identification and analysis of novel genetic variants associated with weight gain (54). The genetic determinants of polygenic obesity in children have been reported in **Table 2**.

**Table 2:** Genetic determinants of polygenic obesity in children.

GENE NAME	GENE’S PRODUCT FUNCTION	MUTATION	PHENOTYPE	REFERENCE
**Fat-mass and obesity-associated gene (FTO)**	RNA demethylase that mediates oxidative RNA demethylation that acts as a regulator of fat mass and energy homeostasis	Variants: - rs9939609 (intronic variant) - rs9930506 (intronic variant) - rs1421085 (intronic variant) - rs8050136 (intronic variant)	Overweight and severe obese phenotype in different pediatric populations	[Frayling TM, 2007; Wardle J, 2008; Cecil JE, 2008; Rutters F, 2011; Jacobsson JA, 2008; Mangge H, 2011; XI et al., 2010; Todendi PF, 2020; Reuter ÉM, 2021] Scuteri A, 2007 [Grant SF, 2008; Mejía-Benítez A, 2013, Albuquerque D 2013; Inandiklioğlu N and Yaşar A, 2021]
**Melanocortin receptor 4 (MC4R)**	Energy homeostasis, food intake and body weight regulator	rs17782313	Obesity traits and metabolic phenotypes in Portuguese school children	[Almeida SM, 2018]
**Tumor necrosis factor (TNF)-α**	Pleiotropic cytokine, important mediator of inflammation	rs1800629	Males Portuguese obese adolescents Normal weight Romanian children	[Nascimento H, 2016; Mărginean CO, 2019].
**Interleukin (IL)-6**	Pleiotropic cytokine important in regulating immunological and inflammatory responses	rs1800795	Obesity in Egyptian children	[Ibrahim OM, 2017]
**Long noncoding (LncOb) RNA**	Regulator of white adipogenesis	rs10487505	BMI and leptin levels in Italian children	[Manco M, 2022]

Among the obesity-related genetic variants, those located in the fat mass and obesity associated (FTO) gene locus, were the first ones that showed a strong association with BMI. The FTO gene, expressed in the hypothalamus, is located on chromosome 16 and encodes a demethylation enzyme implicated in the control of energy homeostasis, food intake and energy expenditure (55). In particular, two studies demonstrated that the variants rs9939609 and rs9930506 in the first intron of the FTO gene are significantly associated with BMI, thus suggesting that the presence of these variants may increase the risk of obesity in both adults and children (56, 57). Accordingly, the rs9939609 variant was found to be associated with increased fat mass and BMI in Scottish children (58) and to obesity phenotype in Dutch children (59). A further study reported that the rs9939609 allele may predispose to pediatric obesity risk by acting on satiety responsiveness (60). An interesting study by Jacobsson et al. highlighted the rs9939609 variant was associated with obesity and gender in Swedish children (61). Moreover, in European adolescents with obesity the homozygosity for the rs9939609 FTO SNP was critically associated with trunk-weighted (62). Besides in the Caucasian population, the presence of the common risk allele of rs9939609 polymorphism has been significantly associated with BMI and obesity in large population-based survey performed in Chinese children, where the polymorphism was associated with weight, BMI, waist circumference, waist-to-height ratio and fat mass percentage (63). A meta-analysis collected and analyzed all these studies suggesting that the rs9939609 has to be considered as the major FTO gene variant associated with overweight/obesity across multiple pediatric populations (64). However, studies on the impact of rs9939609 variant were performed also in Brazilian children and adolescents (65, 66). Todendi et al. reported a correlation between AA genotype for the rs9939609 variant and alteration of waist circumference and overweight/obesity in both children and their parents, thus corroborating the parental contribution to obesity onset (65). A more recent longitudinal study by Reuter et al. have confirmed the association of rs9939609 variant and abdominal fat in Brazilian school children between 7 and 15 years old (66).

Of note, a line of evidence showed no association between FTO rs9939609 and BMI, thus highlighting that the ethnicity may be pivotal to determine the role of this variant on obesity risk (67–69). Other two FTO gene variants, the rs8050136 and the rs1421085, were found associated to obesity traits in different children population studies alone or in combination to the best investigated rs9939609 variant (67, 70–72). Although, the true role of these additional loci remain unknown, deserving further investigations in the next future, a very recent meta-analysis reported this and other variants in FTO genes and their association with pediatric obesity overall (73). Often FTO variants have been investigated with variants in the MC4R of which mutations can be positioned between monogenic obesity and the polygenic obesity (36, 73, 74).

Since there are several published studies reporting the critical pathogenic role of pro-inflammatory cytokines in obesity, further genetic association studies were focused on the influence of variants in genes encoding for these molecules in obesity (75). In obesity, increased production and secretion of a wide range of inflammatory molecules, including tumor necrosis factor (TNF)-α and interleukin-6 (IL-6) was reported in white adipose tissue (76). TNF-α was found to be involved in lipid metabolism leading to hypertriglyceridemia as a result of decreasing lipoprotein lipase activity and increasing hepatic *de novo* synthesis of fatty acids, while IL-6 is mainly associated with liver and adipose tissue inflammation (77). The rs1800629 variant in the TNF-α gene, associated with increased expression of the cytokine in adipose tissue, was found to be more frequent in obese children than in lean subjects (78, 79). However, to date the role of TNF-α rs1800629 on childhood obesity is not clear. A study on Portuguese obese adolescents revealed that only in males subjects, the circulating TNF-α levels were modulated by TNF-α rs1800629 (80). Instead, a study on Romanian children (aged 5–18 years), revealed that TNF-α rs1800629 variant was more frequent in normal weight than in overweight children (81). Few studies demonstrated that also a variant in IL-6 gene may correlate with the obese phenotype (82). In particular, the IL6 polymorphisms designated as rs1800795 as well, were associated with the risk of developing obesity in Egyptian children (83).

The mentioned SNPs only explain 6% of BMI elevation, therefore Khera et al., by incorporating the analysis from 2.1 million SNPs, proposed a polygenic risk score (PRS) that increased the prediction of BMI alteration up to 23%. Interestingly, this PRS was associated with adult BMI, but it showed a consistency in children too (84).

Recently, a GWAS identified nineteen significant genome regions associated with body fatness (85). From this study emerged, for the first time, the association of BDNF, Fragile Histidine Triad Diadenosine Triphosphatase (FHIT), and protein kinase D1 (PRKD1) gene regions with BMI in children.

Growing evidence showed that genetic variation (typically SNPs) can also affect epigenetic profiles independently of or in contribution with environmental factors. In particular, have been identified several variants in methylation quantitative trait loci (meQTLs) that are differentially distributed across the genome and are involved in obesity and metabolic traits (86). A meta-analysis showed that the rs17782313 variant may regulate the expression of MC4R gene by affecting its methylation levels, thus contributing to childhood obesity (87). Further studies by using the analysis of meQTLs with GWAS may help in understanding how genetic predisposition and environmental factors interact with each other to determine methylation status that predispose to develop an obese phenotype in children. Moreover, future research should attempt to verify whether methylome signals of prenatal exposure can be regulated by genetic variation/specific single polymorphisms in metabolic genes correlated also with children and adolescents’ obesity thus confirming the possibility of both a passive and active role of DNA methylation to regulatory interactions influencing gene expression (86, 88, 89).

Finally, very recently a group of studies have reported the relevance of rs10487505 variant of the region encoding for the long non-coding RNA lncOb, which was correlated with leptin levels and BMI in adults and children (90, 91).

## Pediatric obesity epigenetics

Epigenetic changes in the human genome are defined as heritable regulatory mechanisms of gene expression overlaying the information enclosed in the DNA sequence. Epigenetic changes (also referred to as epigenetic marks) include mainly DNA methylation, post-translational modifications of histones, and ncRNAs (92, 93). DNA methylation, which is the most studied epigenetic mark, is a process occurring on the fifth carbon of cytosines mostly located in regions with symmetrical CpG dinucleotides, where the addition of a methyl group in the cytosine may cause transcriptional repression. This process is sustained by the activity of three types of DNA methyltransferases (DNMTs), including DNMT1, DNMT3A and DNMT3B (92). The term of post-translational modifications of histones comprises of different chemical changes, such as histone acetylation, histone methylation, histone phosphorylation, and histone ubiquitination. These modifications may be regulated by different enzymes, such as histone acetyltransferases, histone deacetylases, histone methyltransferases and demethylases, and may be associated with both transcriptional activation and repression (93). Commonly, ncRNAs are a group of RNAs that are classified according to their length in long ncRNAs (lncRNAs), and small ncRNAs including miRNAs, small nucleolar RNA (snoRNA), small nuclear RNA (snRNA), PIWI-interacting RNAs (piRNAs), and others, which may regulate gene expression at transcriptional, post-transcriptional, translational, and post-translational level in a variety of ways (94, 95).

There are several environmental and dietary factors (i.e. overnutrition) that may influence epigenetics, thus acting as regulators of the environment-gene interaction over the life course (96). A recent updated systematic review emphasized the association between epigenetic mechanisms and obesity in children (97). This systematic review describes studies reporting strong evidence of an epigenetic signature assessed by Epigenome-Wide Association Study (EWAS) in childhood obesity. Indeed, a recent EWAS by Robinson et al, based on the UK Avon Longitudinal study of Parents and Children (ALSPAC) cohort was able to identify for the first time the association between a differential DNA methylation profiles of blood samples in children with early life rapid weight gain. The candidate gene analysis on ALSPAC and in the replication cohort found a positive association between rapid growth gain and DNA methylation at the locus near the checkpoint with forkhead and ring finger domains (CHFR) (cg11531579) gene encoding for E3 ubiquitin- protein ligase, thus increasing the risk of subsequent pediatric overweight/obesity (98).

Xu et al. showed a substantial number of differentially methylated CpG sites between obese and non-obese adolescents (14–20 years old) and associated to gene previously linked to obesity and T2D, such as FTO, glucokinase (GCK), hepatocyte nuclear factor-1 α and β (HNF1A and HNF1B), peroxisome proliferator-activated receptor gamma (PPAR*γ*), phosphatase and tensin homolog (PTEN), and transcription factor 7-like 2 (TCF7L2) (99). Subsequently, numerous studies have associated DNA methylation patterns with obesity, based on BMI or waist circumference (WC) in both adults and children (100, 101). Moreover, increasing evidence demonstrated that transgenerational inheritance of environmental exposures may be passed down to generations of offspring never exposed to the parental stimuli (12). All these findings support the emerging evidence that pre-pregnancy paternal exposure, and/or exposure during pregnancy to both maternal weight gain and obesogens could explain most of transgenerational epigenetic programming that explain the hereditary nature of obesity and its related comorbidities in children (102–104).

In the next paragraphs, we will report the literature evidence of transgenerational inheritance of epigenetics due to parental overnutrition or *in utero* exposure to factors that may promote an obese phenotype in children.

### Transgenerational inheritance of epigenetics due to pre-pregnancy parental factors

Increasing evidence links parental nutritional status to epigenetic programming in offspring explaining the hereditary nature of metabolic diseases such as obesity, thus supporting the emerging concept of DOHaD that emphasizes the role of environmental factors during the embryonic period in determining the development of lifestyle-related disorders in adulthood (105).

**Figure 1:**
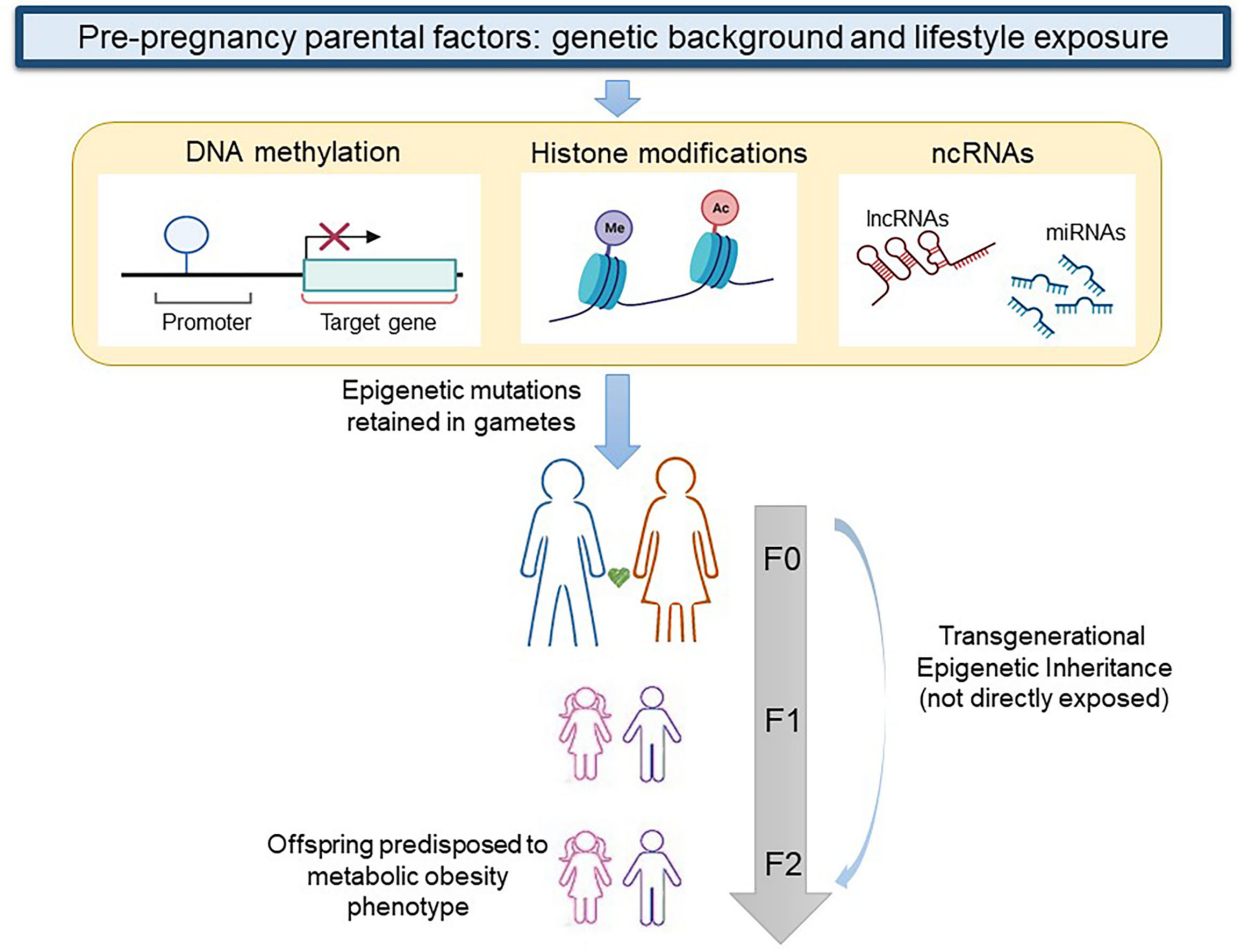
Transgenerational epigenetic inheritance of obesity. When genetic background and lifestyle exposure cause epigenetic modifications (DNA methylation, histone modifications, and changes in non-coding RNAs) in gametes of a woman or a man at F0, the first generation that exhibits a transgenerational epigenetic inheritance is the F2. *lncRNA, long non coding RNA; ncRNA, non coding RNA; miRNA, micro RNA.* Figure partially created with BioRender.com.

In the transgenerational inheritance the insult may alter the germline epigenomic signature during preconception and transmit the epimutations to the F2 generation without any exposure as shown in **Figure 1** (106). One of the first studies that reported evidence on the role of human pre-conception parent nutritional status on epigenetic inheritance examined the Dutch famine birth cohort and its offspring. In particular, the study demonstrated that offspring of exposed F1 fathers were heavier and had a higher BMI than offspring of unexposed F1 fathers (107). Recent studies especially in animal models have demonstrated that both obesity and diabetes changed levels of DNA and histone methylation, histone acetylation, and ncRNAs, such as miRNAs in oocytes and sperm (108). In particular, it is well known that DNA methylation reprogramming is a crucial event that takes place immediately post-fertilization and during gametogenesis (109). DNA methylation was analyzed at imprinted regions which include genes with monoallelic expression and for this reason susceptible to the paternal or maternal modulation in the progeny. Insulin like growth factor 2 (IGF2) gene and H19 lncRNA are well-described imprinted genes associated with childhood obesity which are subject to epigenetic regulation. The IGF-2 gene is a paternally imprinted gene encoding the protein IGF-2 that presents a high affinity with the insulin receptor and is related to mitogenesis and mitochondrial biogenesis. On the other hand, H-19 is a maternally imprinted gene that also is related to muscle insulin sensitivity (110, 111).

Unfortunately, the effect of epigenetic changes occurring in germline that may influence progeny obesity are poorly investigated because human sperms and oocytes are not available for research studies. Therefore, the negative effect on oocytes, and the link between obesity and epigenetics, have been performed mainly in animal models as reviewed by Ou et al. (108). In particular, animal models of diet, such as high-fat diet (HFD), or genetic-induced (i.e. ob/ob mice) obesity were found characterized by altered levels of DNA methylation in the oocytes (112, 113). HFD obese and ob/ob mice also exhibited a reduction of expression of STELLA protein (i.e., a maternal gene required for oogenesis and early embryogenesis) in oocytes that affected the levels of DNA methylation (114). Moreover, in the same experimental model Hou et al. reported modifications in the methylation levels of histone H3 lysine 9 and lysine 27 (113). Studies on ncRNAs and oocytes from individuals with obesity are still lacking.

Experimental studies by animal models also demonstrated a link between paternal obesity and changes in sperm cells DNA methylation profiles (112, 115). However, the interest for epigenetic inheritance of chronic metabolic disorders through the human paternal germ line is recently growing (116, 117). A previous human study demonstrated that babies born to fathers with obesity have altered DNA methylation at several regulatory regions of imprinted genes compared with children born to parents without obesity. The analysis of DNA methylation percentages at the differentially methylated regions (DMRs) from 92 umbilical cord blood leukocyte samples of newborns revealed that paternal obesity was significantly associated with lower methylation levels at the mesoderm specific transcript (MEST), paternally expressed 3 (PEG3), and neuronatin (NNAT) genes important for normal growth and development, suggesting the susceptibility for the next generation to develop chronic diseases in adulthood (118). Interestingly, a human study which compared sperm DNA methylation percentages, at 12 DMRs in 23 overweight/obese and 44 normal weight men showed that the obesity status is traceable in the sperm epigenome (119). In particular, the study reported a significant reduction of methylation levels in maternally expressed 3 (MEG3), necdin (NDN), small nuclear ribonucleoprotein polypeptide N (SNRPN), and epsilon sarcoglycan (SGCE)/paternally expressed 10 (PEG10) genes and a slightly increased of DNA methylation at the intergenic MEG3 and imprinted maternally expressed transcript (H19) DMRs in overweight/obese subjects compared to normal weight men (119). Recently, the impact of male obesity on DNA methylation reprogramming in sperm was also investigated by Keyhan et al. The author identified 3264 CpG sites in human sperm that are significantly associated with BMI in mature spermatozoa, then remarking the potential heritability of these DNA methylation alterations to the next generations (120).

Current research on how spermatozoa transmit acquired epigenetic changes affecting the metabolic health of the next generation revealed that both high-fat diets and low-protein diets may cause changes in spermatozoal content of ncRNA subtypes. In particular, altered sperm levels of snRNA due to high-fat diets in particular were associated with an altered phenotype in the offspring, with the offspring showing insulin resistance, altered body weight, and glucose intolerance (121). In men with obesity it was also observed an altered expression level of specific miRNAs, piRNAs, tRFs, and small nuclear RNA (snRNA) fragments in the spermatozoa with respect to lean men (122). Moreover, the authors reported also changes in DNA methylation patterns between obese and lean men in some genes, including neuropeptide Y (NPY), cannabinoid receptor type 1 (CR1), cocaine- and amphetamine-regulated transcript (CART), FTO, and carbohydrate sulfotransferase 8 (CHST8). Interestingly, the sperm pattern of DNA methylation was modulated by bariatric surgery, highlighting the relevance of the impact of weight loss and nutritional intake on the epigenome of human sperm (122).

Tissue and circulating miRNAs were extensively studied in the context of pediatric obesity (123, 124) but there are no studies on their pre-pregnancy modulation and effects on the obese phenotype on offspring. A work by López and colleagues showed that two miRNAs associated with inflammation and iron homeostasis (miR-155 and miR-122) were upregulated in sperm cells of obese subjects compared to subjects with normal weight (125). These findings support the hypothesis that differentially regulated microRNAs in sperms may potentially act upon DNA methylation post fertilization.

### Epigenetic effects of *in utero* exposure to maternal obesity/overnutrition and environment

**Figure 2:**
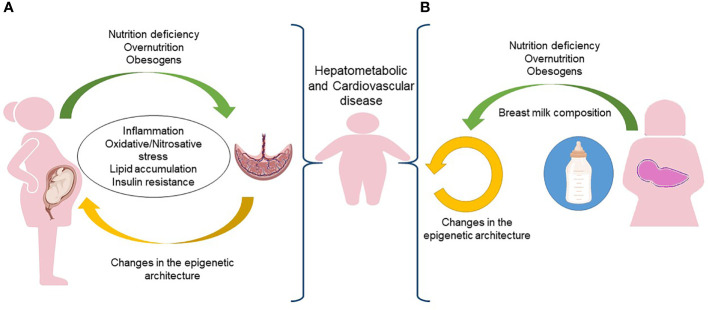
*In utero* and postnatal predisposition to obesity. **(A)** During pregnancy, the placenta of a woman exposed to nutrition deficiency, overnutrition or obesogens, is subjected to inflammation, oxidative/nitrosative stress, lipid accumulation and insulin resistance that increases the risk of obesity and its comorbidities in the newborn. **(B)** The same risk of obesity and comorbidities early in the life could be associated to epigenetic changes occurring in the breast milk. Figure partially created with BioRender.com.

The interface between mother and fetus is the placenta, which regulates the flow of nutrients, oxygen, and hormonal supply. It follows that the placenta is involved in any abnormalities of fetal growth and of the resulting hepatometabolic and cardiovascular diseases in the offspring (126, 127). As depicted in **Figure 2A**, the placenta of pregnant women with obesity is subjected to increased inflammation and oxidative stress (128). Several data suggest that the chronic low-grade systemic inflammation in obese pregnancy and the accumulation of macrophages in the placenta cause uncontrolled local inflammation due to the production of pro-inflammatory cytokines that may alter maternal nutrient transport (129). A nonhuman primate model of excess nutrition in pregnancy provides evidence that a HFD decreases uterine blood flow and causes placental ischemia (130).

In 2015 Sharp et al. analyzed the Avon Longitudinal Study of Parents and Children including a general population pregnancy cohort found that maternal pre-pregnancy BMI was positively associated with cord blood methylation at two CpG sites, at regions encoding for coiled-coil domain-containing 112 (CCDC112) and mucolipin TRP cation channel 3 (MCOLN3). Moreover, offspring of women with obesity had 28 CpG sites identified that were differentially methylated compared with offspring of women with normal weight. Four CpG sites in children peripheral blood were associated to maternal obesity. The study also reported a stronger association between offspring methylation and maternal obesity compared to paternal obesity, supporting an intrauterine mechanism (131).

The DNA methylation profile changes in the fetus can be influenced by the amount in the maternal diet of methyl donors from which depends the one carbon metabolism, including folate, methionine, betaine, or choline, thus leading to adverse metabolic effects during infancy and childhood. Maternal choline and betaine deficiencies also alter the methylation status of genes that participate in growth (Insulin-Like Growth Factor 2 Receptor, IGRF2, corticotropin releasing hormone, CRH, and nuclear receptor subfamily 3 group C member 1 (NR3C1) or vascular function (vascular endothelial growth factor C,VEFGC, and angiopoietin 2, ANGPT2), resulting in developmental abnormalities, blood vessel malformation, or increased predisposition for developing steatohepatitis in the offspring (132).

In the last ten years EWAS provided a useful and successful tool for longitudinal study designs of childhood obesity. Several studies highlighted the presence of a significant association between DNA methylation, obesity and some risk factors as birthweight and maternal BMI, supporting the emerging consensus on the heritability of BMI (133, 134).

Methylation status of leptin and its receptor (LEPR) gene promoters is one of the first evidence supporting the idea that epigenetic variations linked with intrauterine exposures and DNA methylation may influence childhood obesity (135). A further study demonstrated that a maternal poor weight gain during pregnancy was associated with increased methylation status of LEPR thus affecting the expression of the protein in umbilical vein of newborns (136).

A recent systematic review reported data from EWAS which investigated the DNA methylation levels, in peripheral blood or cord blood samples of children with or without obesity and/or overweight. Overall, 19 studies out of 23 found an association between childhood anthropometric parameters with at least one methylated CpG site or region. In four genes [histone deacetylase 4 (HDAC4), prolactin-releasing hormone receptor (PRLHR), tenascin XB (TNXB) genes, and PR domain containing 16 (PRDM16)] it was found the association of a DNA methylation profile with childhood obesity (97).

A meta-analysis study concerning data from EWAS of seven cohorts participating in the Pregnancy and Childhood Epigenetics (PACE) consortium found that maternal gestational diabetes mellitus (GDM) was associated with lower cord blood methylation levels within two regions. One region covering the promoter of OR2L13, a gene associated with autism spectrum disorder, and the other one covering the cytochrome P450 family 2 subfamily E member 1 (CYP2E1) gene, which is upregulated in type 1 and T2D (137).

A recent EWAS using maternal blood and cord blood samples from FinnGeDi (Finnish Gestational Diabetes) study cohort (298 mother-offspring pairs with GDM and 238 without) aimed to explore offspring-specific epigenetic effects in response to GDM exposure and maternal methylation levels (138). By a linear regression analysis, the authors found significant association of methylation at the transcription factor CP2 (TFCP2) gene in offspring of GDM exposure and maternal methylation. Furthermore, using the false discovery rate, the authors found seven significant DNA methylation differences positions between the mother’s methylation and GDM status. These loci included: DLG-Associated Protein 2 (DLGAP2) gene, H3 clustered histone 6 (H3C6) gene, family with sequence similarity 13 member A (FAM13A) gene, ubiquitin protein ligase E3C (UBE3C) gene, LOC127841, and two loci within intergenic regions. Interestingly, DLGAP2 and FAM13A, have previously been identified to have a role associated with diabetes and maternal insulin sensitivity in pregnancy, thus suggesting a possible function of their methylation in the future risk of obesity and T2D in offspring (139). Moreover, methylation changes in both FAM13A and DLGAP2 genes in newborns were also associated with environmental changes during pregnancy, including air pollution and smoking in pregnancy, respectively (140, 141).

The role of fetal programming in early infant weight gain due to maternal obesity has been recently further suggested by a study comparing the miRNA profile in peripheral blood mononuclear cell cultures of children from mothers who are normal weight or those who are overweight/obesity. The miRNA profile in mononuclear cells showed a downregulation of miR-155, miR-221, and miR-1301, as well as upregulation of miR-146a in the circulation of newborns from overweight and obese mothers. Moreover, the study showed that in the same samples from newborns, the RNA profile changes induced by overweight mothers and obesity were also associated to alteration in PPAR-γ gene expression, a master regulator of adipogenesis and altered cytokine pro-inflammatory levels *in vitro* blood mononuclear cell response to metabolic stimuli (142).

Also epigenetic regulation of intergenic regions appears to be important for obesity etiology. Indeed, Sasaki et al. (143) have recently shown that genomic loci associated with DMRs localized to intergenic regions and gene bodies most likely influence gene regulation. In particular, pathway analysis revealed that males, had a significant overrepresentation of genes involved in endocytosis and pathways in cancer, including IGF1R, which was previously shown to respond to diet-induced metabolic stress in animal models and in lymphocytes in the context of childhood obesity.

## Parental epigenetic effects on obesity-related comorbidities

DNA methylation, considered as the key epigenetic mechanism by which maternal and paternal exposure to different dietary patterns, toxins or stressors causing phenotypical effects in unexposed future generations, may also explain some of the obesity-related comorbidities (132, 144). Studies reported that maternal obesity or overnutrition during pregnancy and/or lactation can modulate DNA methylation of different genes involved in energy metabolism, glucose homeostasis, insulin signaling and fat deposition, which support the role of DNA methylation in maternal obesity-induced risk of offspring NAFLD, obesity, and diabetes (145).

Several animal studies have shown that maternal nutrients and caloric regimen can impact fetal adiposity, interfering with long term metabolic homeostasis (146, 147). In murine models a HFD during pregnancy causes quite variable effects on fetal birth weight (130). These disparities in results are consistent with human observational studies showing increased rates of either large or small-for-gestational-age infants of mothers with obesity, although still both of these neonatal conditions are associated with risks of early onset childhood metabolic disease (148, 149) and histologically proven NAFLD as well (150, 151). Since obesity related NAFLD in pregnancy is independently associated also with hypertensive complications, postpartum hemorrhage and preterm birth, it should be considered a high-risk obstetric condition, with clinical implications for pre-conception counseling and pregnancy care (152).

In a mouse model, female offspring of mothers with obesity exposed to HFD was affected by obesity with high levels of inflammation in the adipose tissue in association with hypomethylation at certain inflammatory genes not only to the immediate offspring but also over three generations (153). Human studies suggested that social, environmental (e.g. education or cigarette smoking, respectively) and metabolic factors playing a role in the health of grandparents could influence the birth weight, the cardiovascular health, or even the neurodevelopmental outcomes of the grandchildren (154). This potential transgenerational inheritance is therefore now considered a relevant contributing factor to the epidemic levels of obesity and related comorbidities.

De Jesus et al. investigated the impact of paternal and/or maternal metabolic syndrome on the epigenetic reprogramming of metabolic homeostasis in offspring by using a non-dietary, genetically liver-specific insulin receptor deficient mice model of metabolic syndrome phenotype, including hyperglycemia, insulin resistance, and dyslipidemia. The experimental study found that parental metabolic syndrome induces downregulation of growth differentiation factor 15 gene in offspring by modulating the DNA methylation levels at the promoter region of the gene and boosting hepatic lipid accumulation in offspring (155). A study, investigating DMR in nondiabetic Pima Indians who were offspring of diabetic or nondiabetic mothers, supported the hypothesis that epigenetic changes may increase the risk of T2D in the offspring of mothers with diabetes during pregnancy (156). Moreover, a human study involving 128 offspring born at term to mothers with well-controlled GDM highlighted that this condition is associated with changes of DNA methylation at the alternative transcription start site zinc finger protein 696 (ZNF696) in cord blood cells, and changes of plasma glucose levels in newborns at 1 h after birth (157).

All in all, it emerges that the heritable epigenome, such as DNA methylation, histone modifications, and the expression of ncRNAs, could also predispose individuals to metabolic disorders and obesity phenotype. The epigenetic changes could be a target to control antepartum hyperglycemia, prevent gestational diabetes, and avoid excessive weight gain during pregnancy reducing childhood obesity and its related comorbidities. This proposed link between increased maternal insulin resistance during pregnancy and the development of T2D in the offspring should be taken into consideration for actively promoting changes in lifestyle before and during pregnancy in order to prevent the risk of developing obesity and obesity related metabolic diseases (157, 158).

### Effect of maternal nutrition intake during pregnancy

Maternal serum free fatty acids reach also the placenta which represents a site of inflammatory and hypoxic changes. The unfolded protein response/endoplasmic reticulum stress (ERS) is, in fact, a source of reactive oxygen species. Upon disruption of mitochondria-associated membranes integrity, miscommunication directly or indirectly disturbs Ca^2^+ homeostasis and increases ERS and oxidative stress. Obesity-related maternal ERS and oxidative stress reaching the placenta may trigger inflammatory cytokines production and release. These molecules with maternal lipids may reach, through the umbilical cord vein and fetal portal vein, the fetal liver where they start to trigger hepatic lipogenesis and hepatocellular damage (159). In this scenario, the excess of maternal fatty acids could result in the accumulation of fetal ectopic fat. As the fetus subcutaneous adipose tissue (SAT) depot is not yet functioning, the accelerated adipogenesis during the perinatal window of SAT development underlies hepatometabolic and cardiovascular risk in offspring born to dams that are metabolically compromised (**Figure 2**). The exact mechanisms whereby these gestational events exacerbate the pathogenesis of fetal liver damage and also to cardiovascular risk are still poorly understood (150). Animal model studies showed that maternal obesity and HFD have a lasting cardiometabolic impact, and contribute to an increase in fetal liver inflammation and fatty liver in offspring (160–163).

In adult liver it appears quite well-established that NAFLD initiation and progression depend on several events. Among others, up-regulation of sterol regulatory element-binding protein-1c, a transcriptional activator of lipogenic enzymes such as stearoyl coenzyme-A desaturase1 and fatty acid synthase, which contributes to lipogenesis with an increased *de novo* synthesis of fatty acids, represent strong candidates for being involved also in fetal NAFLD (164, 165). NAFLD progression is also reflected by dysregulation of monocyte to macrophage differentiation-associated 2, the death decoy receptor TR6/DcR3 inhibiting T cell chemotaxis *in vitro* and *in vivo*, and chronic inhibition of nitric oxide synthase (166).

### Effect of chemical obesogens during pregnancy

In addition to dietary composition, the exposure to chemical obesogens during pregnancy and early life is another mechanism that can strongly influence the subsequent predisposition to obesity. Obesogens are chemicals that inappropriately stimulate directly or indirectly adipogenesis and fat storage. According to animal models, the epigenetic effects of the exposure may persist on exposed animals and their offspring at least until generation F4 (102).

Exposure to mixtures of phthalates, parabens and other phenols is very common due to their ubiquitous prevalence in personal care products and plastics with also a risk of prenatal exposure. In a longitudinal study of mothers’ prenatal urinary concentration of phthalates and phenols was correlated with the z-score of BMI of the respective children (167).

Also the exposure to plastic derived endocrine disruptors EDCs (e.g. bisphenol) may affect the fetal growth and contribute to epigenetic and angiogenic changes, thus promoting the development of adult chronic diseases. Of note, these compounds may control the processes of epigenetic transgenerational inheritance of adult-onset diseases by modulating DNA methylation and epimutations in reproductive cells leading to the development of obesity and impaired glucose metabolism in the F2 generation (168). The endocrine disrupting effects of obesogens may directly or indirectly promote adipogenesis and obesity/obesity related metabolic conditions that drive metabolic syndrome through disturbance of several processes. Studies show that these effects are seen not only in individuals but also in their offspring because of their ability to epigenetically reprogram genetically inherited set points for the control of body weight and body composition at some critical stages of development, such as fetus, early life, and puberty (169, 170).

### Epigenetics as a target for prevention and therapy of obesity and its related comorbidities

Promoting healthy early programming of adipose tissue is essential to implement new strategies that can control the spread of obesity and other metabolic disease (171, 172). However, the same obesogenic environment may have different effects on different individuals, highlighting an underlying individual susceptibility to obesity and fat distribution (173). In this scenario, the reversal of epigenetic changes could be used as an early and virtuous clinical intervention for reducing the risk of obesity in offspring (171). This goal is achievable through the adoption of healthy eating habits before and during critical periods of development, such as pregnancy and lactation (174). The particularly sensitive period capable of implementing epigenetic reprogramming strategies does not include only pregnancy, neonatal and infant life but encompasses a longer period defined the period of 1000 days, starting from conception and lasting the first 2 years of life (175). The milk of each mother is specific to her own baby, and the maternal-fetal dyad may affect the composition of breastfeeding and the baby’s tolerance to biologic breast milk (176). The identification of factors inducing epigenetic changes in breast milk, have led to the hypothesis of the possibility of transferring epigenetic information directly through breast milk (**Figure 2B**). Currently, studies in humans discovered more than 2000 miRNAs that can regulate collectively one third of the genes in the genome. The human milk provides abundant amounts of miRNA-148a, miR-152, miR-29b, and miR-21, which all target DNA methyl transferases (177, 178). The concept of milk siblings has been known for years (179, 180) and on this basis a study on rats showed that adoptive siblings, who had taken the same milk, had acquired the same hereditary epigenetic heritage (miRNAs). As in the consanguineous marriages, the children born from the marriage of these “milk siblings” may develop more serious pathologies (e.g., differentially expressed miRNAs were associated with pathways regulating metabolism, survival, and cancer development insulin signaling pathways).

Interpreting these results, we can deduce that the changes and strategies (nutrition, physical activity, and no-smoking) adopted during pregnancy and lactation can reverse the trend of a previous metabolic risk in the offspring. The lack of effective drugs easily available for the treatment of obesity and its comorbidities, such as obesity related-NAFLD, requires approaches with very timely changes in lifestyle (i.e. less sedentarism, more sleeping hours and exercise) and better eating habits. It has been shown that a normal pre-pregnancy maternal BMI and long duration of exclusive breastfeeding are favorable conditions not only for a direct effect on weight control, but also to prevent the development of NAFLD later in life (175).

Due to the lack of convincing data of efficacy and safety for both pregnant women with obesity and their offspring after pharmacological interventions during gestation with available medications (i.e. metformin) (181–184), the epigenetic modifiers may represent a promising approach (145, 185). Interestingly, maternal diet supplemented with methyl donors during lactation has been shown to prevent the development of an obese phenotype and fatty liver of offspring in mice (186). Similarly, recent studies in humans found that both paternal and maternal diet supplemented with choline, betaine, folate, and methionine, can affect offspring’s DNA methylation of genes involved in the regulation of metabolism, growth, and appetite (187, 188).

Although these findings suggest that a substantial component of obesity and metabolic disease risk has a prenatal developmental basis, the question remains unsolved because its complexity. In fact, in human studies it appears that there is no simple correlation between maternal intake of methyl group donors, higher or lower offspring DNA methylation and subsequent anthropometric outcomes (188). These effects could depend on the different tested developmental ages (170, 188). Alternatively, it has been suggested that the involvement of adjacent or nearby CpGs within the same promoter showing different strengths of association with child’s adiposity may imply highly specific changes in the transcriptional regulation of these genes induced by the developmental environment, rather than generalized changes in promoter methylation (170, 188). All in all, it appears that epigenetic reprogramming may represent an adaptable response through which the developing offspring modify their physiology to the postnatal environment (189).

## Discussion and conclusions

The growing number of studies in the last few decades makes it clear that obesity is influenced by complex interactions between genetic, developmental, and environmental pressures. All these factors in turn create a network of relationships which are the basis for the concept of the multifactorial pathogenesis of obesity and its comorbidities.

It is obesogens evident that genetic factors play a relevant role, even though it has to be well dissected.

Purely monogenic forms of obesity presenting with severe early-onset obesity associated with hyperphagia and endocrine disruption in fact are so rare that the obesity epidemic cannot depend on single gene disorders. Therefore, the interaction of a more complex (polygenic) genetic background with the environment, where hundreds of SNPs exhibiting a heritability pattern similar to other complex traits and diseases can contribute to the development of the obese phenotype, is a more plausible explanation (20, 51).

Moreover, since environmental influences may cause epigenetic changes already in the gamete cell stage of both parents, but also during pregnancy and the early postnatal period including lactation with a lasting effect on the metabolism of the offspring, some inherent considerations follow. First, it appears now paramount the need of implementing the lifestyle and health status of both parents very early, even before conception (160, 190). Second, specific supplementation of the maternal diet with methyl donors during early gestation and during breastfeeding could prevent the development of an obese phenotype and fatty liver of offspring (186, 188). Further studies in this regard are however necessary to clarify relevant issues such as timing and dosage of methyl donors supplementation in pregnant and breastfeeding women (188, 191, 192).

However, the entire scenario of how parental diet and/or exposure to hormones and other environmental factors (e.g, endocrine disruptors) act transgenerationally on the epigenetic landscape and consequently on development of the offspring’s human adipose tissue and its plasticity over the life course requires further investigation (13).

Currently, epigenetic changes appear to be correctable by targeted interventions such as body weight reduction through virtuous dietary changes, exercise, or even metabolic surgery, all of them regularly affecting DNA methylation. However, targeted interventions with specific epigenetic modifiers to reprogram epigenetic changes is an extremely interesting future playground which has already started to be explored in some other fields. Cancer studies have in fact shown that a number of natural phytochemical substances (e.g. polyphenols) can target the epigenetic machinery with potential benefit due to reverting effects in the epigenetic modifications of relevant genes and restoring their protein expression levels (193). Similar new frontiers studies of cells reprogramming will likely prove meaningful if applicable to adipocytes also in obesity and related conditions as well.

In conclusion, although more evidence is needed, the reported literature strongly suggests that the analysis of the epigenetic architecture at the interface between gene expression and the epigenetic environment could be relevant for a better understanding of obesity and its associated comorbidities. Moreover, further studies addressing the potential intertwining between genetic and epigenetics in childhood obesity could provide the hint to improve prevention and therapeutic management.

## Author contributions

NP, CM, AA, and PV contributed to the conception and design of the manuscript. All authors wrote sections of the manuscript and contributed to design of Figures and Tables. All authors approved the submitted version.

## Funding

This study was supported by 5‰ 2022 assigned to AA by the Italian Ministry of Health.

## Conflict of interest

The authors declare that the research was conducted in the absence of any commercial or financial relationships that could be construed as a potential conflict of interest.

## Publisher’s note

All claims expressed in this article are solely those of the authors and do not necessarily represent those of their affiliated organizations, or those of the publisher, the editors and the reviewers. Any product that may be evaluated in this article, or claim that may be made by its manufacturer, is not guaranteed or endorsed by the publisher.
